# Socioeconomic Differences in Cigarette Smoking and Alternative Tobacco Product Use Among Adolescents in a School-Based Smoking Preventive Intervention: Findings From the Second Year of the X:IT II Study

**DOI:** 10.3389/fpubh.2022.825585

**Published:** 2022-02-21

**Authors:** Simone Gad Kjeld, Lisbeth Lund, Susan Andersen, Lotus Sofie Bast

**Affiliations:** National Institute of Public Health, University of Southern Denmark, Copenhagen, Denmark

**Keywords:** smoking, tobacco, snus, e-cigarette, waterpipe, alternative tobacco products, school-based intervention, prevention

## Abstract

**Background:**

Health interventions may differently impact adolescents from diverse backgrounds. This study examined whether a smoking preventive intervention was equally effective in preventing cigarette smoking and use of alternative tobacco products (ATPs, i.e., snus, e-cigarettes, and waterpipe) among students from different socioeconomic backgrounds, i.e., occupational social classes (OSC).

**Methods:**

Data was from the school-based intervention X:IT II targeting 13- to 15-year-olds Danes. The intervention focused on three main components: smoke-free school time, smoke-free curriculum, and parental involvement. In total, 46 schools were included at baseline (*N* = 2,307, response rate = 86.3%). Using a difference-in-differences approach, changes in current smoking and ever use of ATPs were estimated among students in high versus low OSC at second follow-up. Analyses were based on available cases (*N* = 826) and multiple imputations of missing data at the second follow-up (*N* = 1,965).

**Results:**

At baseline (age 13), 1.0% of students from high OSC and 4.8% from low OSC currently smoked cigarettes, while this was the case among 24.5 and 25.6%, respectively, at the second follow-up (age 15). Estimates indicated that social inequalities in current smoking diminished over time (*p* < 0.001). Regarding ATPs, 10.0% of high OSC students and 13.9% of low OSC students had ever used ATPs at baseline, while at second follow-up, 46.8 and 60.8%, respectively, had ever used ATPs. Estimates indicated that social inequalities in ever use of ATPs widened over time (*p* < 0.001).

**Conclusions:**

The X:IT II intervention seemed to diminish socioeconomic disparities in smoking over the study period. Meanwhile, social inequalities in ever use of ATPs increased. Therefore, besides focusing on narrowing the social disparities in cigarette smoking, future efforts may, to a larger extent, focus on adolescents' use of ATPs.

## Introduction

Smoking remains one of the leading causes of years of life lost and increased morbidity in Denmark and worldwide (1, 2). Globally, a critical political and public health issue is protecting children and adolescents from the health hazards of using tobacco products (3). Individuals initiating a tobacco use in their teenage years are more likely to continue smoking in adulthood, be addicted to nicotine, have issues with smoking cessation, and have higher risks of health adversities in later life (4–6). One significant concern is adolescents' being exposed to smoking in their environment, i.e., among parents, siblings, teachers, or peers (7–9). Specifically, when adolescents see others smoke in their near environment, they are more prone to perceive smoking as socially acceptable and susceptible to smoke themselves. Moreover, second-hand exposure to smoke is considered a health risk in itself (1). Therefore, preventing smoking uptake in adolescence and limiting the exposure of smoking in adolescents' everyday lives may have crucial public health benefits. In Denmark, a continuous goal by stakeholders has been to create a smoke-free future for Danish children (10). For several decades, overall smoking prevalences have decreased, e.g., among Danish schoolchildren, daily smoking had decreased from 18.6% in 1991 to 4.5% in 2014 (11). However, more recent reports indicate changing smoking patterns among the youth population; some numbers show a stagnation in smoking uptake among some parts of the Danish youth (12), while others show decreasing trends in daily but not in occasional smoking (13). In Denmark and internationally, there seems to be a stagnation or even an increase in smoking prevalence among youths from lower socioeconomic backgrounds (11, 14). Additionally, social disparities in smoking have not diminished, quite conversely, research indicates increasing socioeconomic differences in smoking during the last decades (11, 15). Consequently, targeting the socioeconomic differences in smoking is essential in smoking preventive efforts. The social inequalities in tobacco-related harms have also been well-documented internationally (16).

Another increasing public health concern is the use of other tobacco products than conventional cigarettes, including e-cigarettes, smokeless tobacco (e.g., snus), and waterpipe (i.e., alternative tobacco products; ATPs). These products have gained growing interest in a Danish and global context—especially among the youth (17, 18). In fact, there has internationally been an increasing trend in youth use of e-cigarettes, snus, and waterpipe in recent years (18–21). In e.g., USA and Norway, the use of some ATPs has even exceeded the use of conventional cigarettes (22–24). However, the research on socioeconomic differences in the use of ATPs is somewhat inconsistent and limited, with some research indicating a social gradient in the uptake of ATPs among students from different socioeconomic groups (18, 25), while other studies found no socioeconomic differences in the use of ATPs (26–28). Several concerns are linked to the increasing use of ATPs, including health risks and the risk of becoming addicted to nicotine (29, 30). Moreover, adolescents may learn rituals associated with cigarette smoking by using, e.g., e-cigarettes, including the body language, taking smoking breaks, and how to handle a tobacco product (31). Another concern is the concurrent use of multiple tobacco and nicotine products—or that using one tobacco product use may impact decisions to use or try other products. For example, a Norwegian study found that using smokeless tobacco in youth increased the risk of smoking conventional cigarettes in adulthood (32). In this connection, there are concerns that ATPs may be a gateway to conventional cigarette smoking (31, 33), while other studies discuss the common liability hypothesis as a possible explanation for using multiple tobacco products and shifting from one tobacco product to another (34). Given these concerns, tobacco control policies aiming at preventing smoking should focus not only on conventional cigarettes but also on the use of ATPs.

Internationally, a host of interventions have been implemented to reduce smoking uptake among adolescents—many in the school setting—with mixed evidence of their effect (35). One study analyzed data from 49 randomized controlled trials on the effect of smoking preventive interventions (including data from approximately 140,000 schoolchildren) and found a 12% reduction in smoking initiation among children in the intervention groups compared with children in the control groups at more than 1 year follow-up from baseline (35). However, they found no effect at 1 year or less. In a Danish context, one of the first smoking preventive interventions which were found to decrease smoking uptake among adolescents was the X:IT study (36). An evaluation of X:IT indicated that if all intervention components were implemented as intended, X:IT could reduce the proportion of adolescents who smokes up to 25% at 1 year follow up (37). Studies evaluating the effect of preventive interventions on other tobacco products than cigarettes are still very sparse, and research with this aim has been called for in recent years (38).

The effect of school-based smoking and health interventions have been found to differ according to adolescents' socioeconomic backgrounds, although the directions are not unidimensional; some research indicated better intervention effects among students from higher socioeconomic backgrounds (39), while other research found that adolescents from lower socioeconomic backgrounds received the highest benefits of health interventions (40). Consequently, health interventions may both widen or narrow social inequalities in health. In the Danish X:IT study, the process evaluation of the intervention indicated that some aspects of the intervention were more easily adopted by students and parents from higher socioeconomic backgrounds (41). These findings may have important implications for the overall effects of the intervention. To our knowledge, no studies have evaluated socioeconomic differences in the trajectories of ATP use before and after implementing a tobacco preventive intervention.

Considering the limited research evaluating socioeconomic differences in tobacco use after the implementation of a smoking preventive intervention, the current study sought to examine trajectories of cigarette smoking among adolescents participating in a school-based smoking preventive intervention: The X:IT II intervention. Further, as a secondary aim, this study examined trajectories in ATP use across socioeconomic groups during the period. Adolescents were followed over the course of 2 years—from baseline (beginning of grade 7; ~13 years) to second follow-up (end of grade 8; ~15 years).

## Materials and Methods

### The X:IT II Intervention

X:IT II is a school-based intervention with the aim of preventing smoking uptake among students from 7th to 9th grade (13- to 15-year-olds). The intervention consists of multiple initiatives to reduce youth smoking and is inspired by previous successful smoking preventive interventions from Norway and Sweden (42, 43). X:IT II is a modified version of the first X:IT intervention developed in 2010 by the Danish Cancer Society and was evaluated in a cluster-randomized controlled trial (RCT) as well as with a qualitative process evaluation (18). Findings from the RCT showed that students at intervention schools had significantly lower odds of smoking cigarettes compared to students at control schools (OR: 0.61, 95% CI: 0.45–0.82) 1 year after implementation of the intervention (19); thus, the evaluation showed overall positive effects in reducing smoking among students. However, the process evaluation indicated that some of the intervention components (i.e., wording and pictures used) were more easily adopted by students and parents from higher socioeconomic backgrounds (22). Therefore, to address these social inequalities in the adoption of the X:IT intervention, a modified version of the intervention was developed—the X:IT II intervention. The three main intervention components of the X:IT II intervention were (44):

- *Smoke-free school time*. At the time of the study, Denmark had a much more lenient smoking policy compared with other Scandinavian countries. In 2007, the first law restricting smoking in public places was employed, and in 2012, smoking at school grounds was fully banned for students, employees, and visitors (45). Smoking rules at X:IT II intervention schools are even stricter than national legislations. Hence, schools included in the intervention are encouraged to ensure that neither students, teachers, other employees, nor visitors smoke during school hours—neither at school grounds nor at other places during school hours, e.g., just outside of the school area, at parks, shops, etc. This is also known as “smoke-free school time.”
- *Parental involvement*. This component consists of two dimensions: 1) smoke-free agreements, which involve that the parent and the child both sign an agreement committing the child not to smoke in the following school year, and 2) smoke-free dialogues between the parent and the child. Here, parents commit to have continuous dialogues with their children about smoking and tobacco use. Parents could receive help for these chats using the website (www.snakomtobak.dk, in English: Chat about Tobacco) developed for the purpose. The website targets several groups of parents, including smoking and non-smoking parents as well as parents with and without children who smoke. At parent–teacher meetings at the beginning of the school year, teachers introduced the website and the X:IT intervention to parents.
- *Smoke-free curriculum*. The educational material “Up in Smoke” (www.opiroeg.dk) was specifically developed to teach students in 7th to 9th grade about smoking, health risks, the pressure of smoking, etc. The material was based on self-efficacy training and appraisal of outcome expectations and included eight lessons a year over the course of 3 years. It was intended to be cross-curricular and, thus, could fit in with ordinary school activities to prevent excess workload on the teachers. In the revised X:IT II intervention, the general readability of the material was improved, and the automated readability index was lowered, e.g., a glossary appears when clicking on academic words such as “cancer” or “oxygen.”

The participating schools had each assigned a school coordinator, most often a teacher, who coordinated intervention activities at the schools and informed colleagues about the intervention.

### Study Design

**Figure 1:**
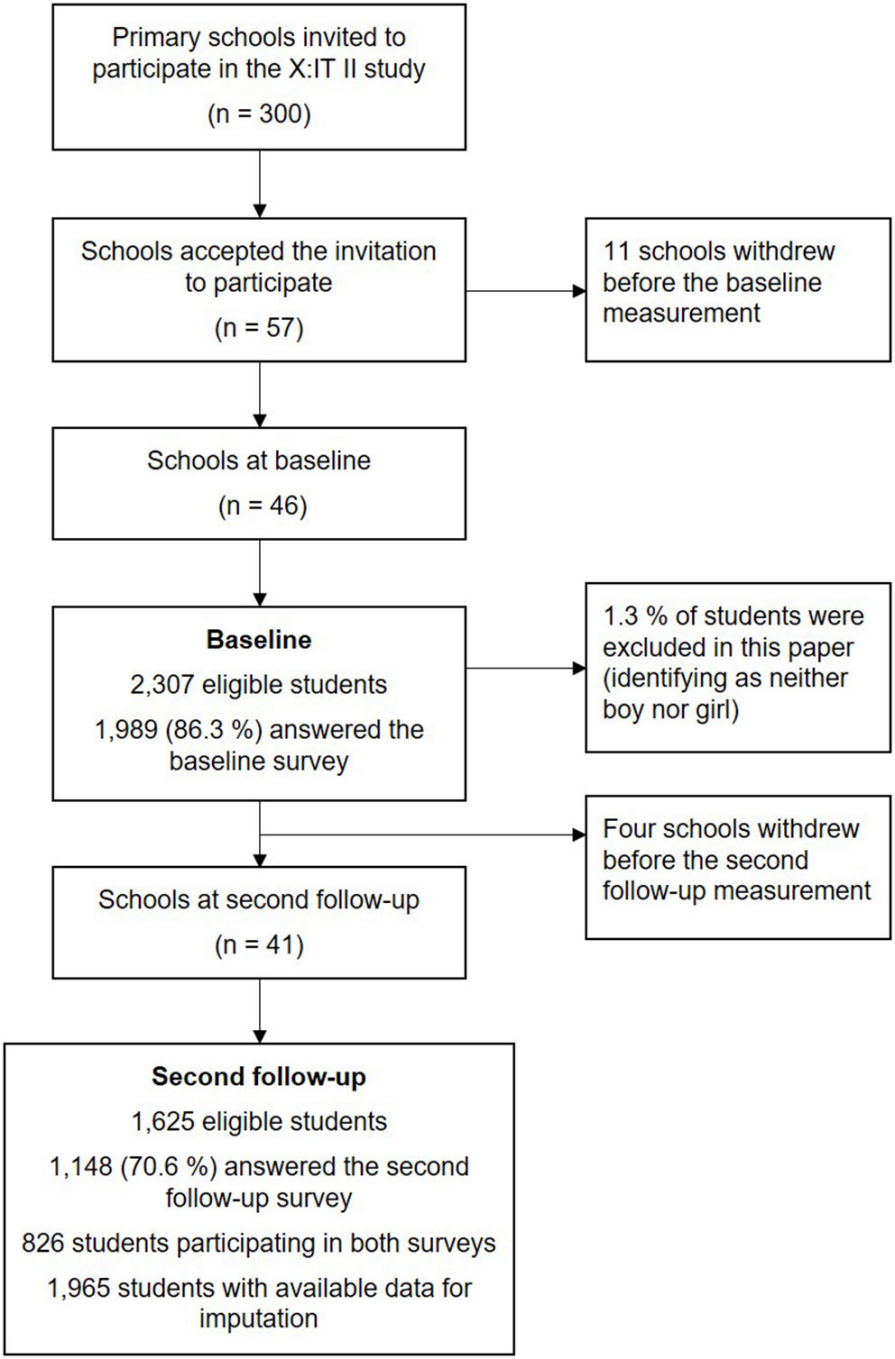
Flowchart of included students in the X:IT II study at baseline and second follow-up.

This study evaluates the X:IT II intervention after the second year of implementation. The intervention was evaluated over the course of 3 years, from 2017 until 2020. Overall, 300 schools were randomly selected and invited to participate in the evaluation of which 57 schools accepted the invitation. However, prior to the baseline data collection, 11 schools withdrew their participation: two schools had hired new school leaders who chose not to prioritize the intervention; two schools had to prioritize other projects because they were involved in more projects at once; and seven schools responded that they did not have time to participate. In total, the baseline measurement consisted of 46 schools across Denmark (Figure 1). Data collection consisted of online self-reported surveys to students and school coordinators at the beginning of 7th grade (baseline) until the end of 9th grade (third follow-up measurement). The school coordinator handled the data collection among students.

This study utilized data from baseline and the second follow-up measurement (at the end of 8th grade in 2019). All students at the 46 enrolled schools were encouraged to participate in the study (*N* = 2,307). Out of all eligible students, 1,989 students (response rate = 86.3%) answered the baseline questionnaire. At second follow-up, 1,148 students responded to the questionnaire out of 1,625 eligible students (response rate = 70.6%). In total, information on both baseline and second follow-up were obtained from 826 students, and 1,965 students had available information in the imputed data set. The current study evaluates the effect of the X:IT II intervention as pre-specified in Current Controlled Trials (ISRCTN31292019).

### Measures

#### Current Smoking

The outcome measure of current smoking was assessed by dichotomizing students' responses to the question “How often do you smoke?” into currently smoking (daily, weekly, monthly, or more seldom) vs. do not smoke.

#### Ever Use of ATPs

The outcome measure of ATP use was assessed by three questions; hence, students were asked if they had ever used one of the following products: snus, e-cigarettes, and waterpipe, respectively. Response categories ranged from a single time and up to more than 40 times. We dichotomized the variable into 1 = had ever used ATPs (i.e., either snus, e-cigarettes, or waterpipe) and 0 = had never used ATPs.

#### Family Occupational Social Class

Socioeconomic position was assessed by two questions about the occupation of students' father and mother (OSC). OSC was coded in accordance with the Danish Occupational Social Class Measurement (46) and was categorized from I = high to V = low social class as well as VI = parents receiving social benefits. The highest-ranking parent determined the OSC. OSC was categorized into three groups: high (I to II), medium (III to IV), and low (V to VI). Previous research has found students to be generally able to answer questions about their parents' occupation status with fair validity (47).

#### Gender

Due to well-established gender differences in smoking uptake and use (48), analyses were adjusted for gender. Gender was assessed with the question “are you a boy or a girl?” with response options 1 = boy, 2 = girl, and 3 = students who felt they did not fit into neither of the two first categories. Students in the last category (1.3% of students) were excluded for further analyses.

### Analyses

A difference-in-differences design was used to evaluate the effect of X:IT II on current smoking and ever use of ATPs according to students' OSC. The difference-in-difference method is considered a useful approach for assessing the impact of interventions by estimating the trajectories of change between two groups before and after receiving a treatment. This method is particularly useful in non-randomized study designs with no obvious control groups (49). Hence, using this approach, trajectories of change in current smoking and ever use of ATPs were estimated before and after the implementation of X:IT II among students in two groups; students from high and low OSC, respectively. High OSC served as the comparison group (group C), while low OSC was considered the exposed group (group E), as the X:IT II intervention was modified to specifically target students from low OSC. Changes in outcomes were estimated as (Cafter–Cbefore)–(Eafter–Ebefore). When difference-in-differences estimates are close to zero, the intervention is expected to be equally effective among students across their OSC. Analyses were based on available cases (*N* = 690), which comprised the proportion of students participating in both the baseline and the second follow-up measurement, and who had responded to the OSC measure as well as measures about tobacco product use at baseline and second follow-up. All analyses were carried out using SAS v. 9.4.

The analyses were based on multiple imputations of missing data at the second follow-up measurement (*N* = 78.600). Imputations of missing data were based on several variables in the baseline dataset commonly associated with smoking (i.e., gender, smoking frequency, ethnicity, OSC, intention to smoke, exposure to smoking at home and school, and parents' attitudes about smoking). First, baseline variables were imputed to ensure a monotone pattern of missing observations in the baseline dataset. Secondly, smoking frequency at second follow-up was imputed using multistage imputation. Because of intraclass correlations between schools and classes, two times 20 rounds of imputations were carried out. First, the school and class effects were overestimated on the standard errors by including the school and class variables as fixed effects in the imputation model. Thereafter, school and class effects were underestimated on the standard errors by not including school and class variables as effects in the imputation model. In total, 40 imputed data sets were created, and as suggested by Graham (50), analyses of these data sets should provide a pragmatic evaluation of the standard error. The PROC MI procedure in SAS was used with a repeated statement for unadjusted analyses and analyses adjusted for gender. The 40 imputed data sets were analyzed with a random-effect logistic regression model including the same variables as used in the analysis of available cases. The results were collected with the PROC MIANALYZE procedure. The same procedure undertaken for the analyses concerning current smoking was used for ever use of ATPs.

Power calculations were conducted in accordance with Donner and Klar (51) based on the following assumptions: each school cluster comprised 50 students; the intraclass correlation coefficient for current smoking among 15-year-olds was 0.053; the smoking prevalence in 9th grade was 17.9%; and the expected reduction in smoking was 25% (i.e., from 17.9% to 13.4%) with a power of 80%. These calculations showed a need for 48 schools comprising around 2,400 students.

## Results

As presented in Table 1A, 826 participants were eligible for analyses who both answered the baseline measurement as well as the second follow-up measurement. In total, 1,139 (58.0%) were considered non-participants, i.e., they did not respond to the second follow-up measurement. More students from a low and medium OSC, who currently smoke, and who have ever used ATPs did not respond to the second follow-up.

**Table 1A:** Characteristics of participants and non-participants in the second follow-up (*N* = 1,965).

	**Participants**	**Non-participants**	*p*-value^*^
	**(*N =* 826)**	**(*N =* 1,139)**	
	**% (*n*)**	**% (*n*)**	
**Gender**			
Boys	47.6 (393)	49.0 (558)	0.537
Girls	52.4 (433)	51.0 (581)	
**Family occupational social class (OSC)**			
High	27.1 (311)	23.7 (362)	<0.001
Medium	26.1 (300)	20.5 (313)	
Low	5.6 (64)	8.4 (129)	
Non-classifiable	12.8 (147)	15.8 (242)	
*Missing*	28.5 (327)	31.6 (484)	
**Currently smoking at baseline**			
Yes	1.3 (11)	2.2 (25)	<0.001
No	98.6 (814)	95.5 (1,088)	
*Missing*	0.1 (1)	2.3 (26)	
**Currently smoking at second follow-up**			
Yes	8.4 (9)	–	–
No	91.5 (756)	–	
*Missing*	0.1 (1)	100 (1,139)	
**Ever use of ATPs at baseline**			
Yes	10.2 (84)	11.7 (133)	<0.001
No	89.7 (741)	84.0 (957)	
*Missing*	0.1 (1)	4.3 (49)	
**Ever use of ATPs at second follow-up**			
Yes	25.3 (209)	–	–
No	74.5 (615)	–	
*Missing*	0.2 (2)	100 (1,139)	

* *p-values estimating differences between participating and non-participating students*.

Table 1B outlines a more comprehensive overview of the participants and non-participants in relation to the use of various ATPs; overall, results show that more students who had ever used waterpipe, snus, and e-cigarettes missed responding at second follow-up.

**Table 1B:** Characteristics of participants and non-participants in the second follow-up, ever use of ATPs (*N* = 1,965).

	**Participants**	**Non-participants**	*p*-value^*^
	**(*N =* 826)**	**(*N =* 1,139)**	
	**% (*n*)**	**% (*n*)**	
**Ever used waterpipe at baseline**			
Yes	4.5 (37)	7.0 (80)	<0.001
No	95.2 (786)	88.2 (1,004)	
*Missing*	0.4 (3)	4.8 (55)	
**Ever used waterpipe at second follow-up**			
Yes	11.9 (84)	–	–
No	87.8 (725)	–	
*Missing*	0.4 (3)	100 (1,139)	
**Ever used snus at baseline**			
Yes	1.5 (12)	1.8 (20)	<0.001
No	97.8 (808)	93.2 (1,062)	
*Missing*	0.7 (6)	5.0 (57)	
**Ever used snus at second follow-up**			
Yes	8.4 (69)	–	–
No	91.0 (752)	–	
*Missing*	0.6 (5)	100 (1,139)	
**Ever used e-cigarettes at baseline**			
Yes	6.5 (54)	7.9 (90)	<0.001
No	93.2 (770)	87.8 (1,000)	
*Missing*	0.2 (2)	4.3 (49)	
**Ever used e-cigarettes at second follow-up**			
Yes	21.9 (173)	–	–
No	78.7 (650)	–	
*Missing*	0.4 (3)	100 (1,139)	

* *p-values estimating differences between participating and non-participating students*.

Table 2 displays descriptive information of baseline cases and imputed cases. Overall, no marked differences are seen between baseline cases and imputed cases in relation to gender as well as current smoking and ever use of ATPs at baseline; however, more students currently smoke (23.8 vs. 8.4%) and have ever used ATPs (49.7 vs. 25.4%) at second follow-up in the imputed cases compared with the baseline cases.

**Table 2:** Descriptive information on baseline cases and imputed cases in the X:IT II study.

	**Baseline cases**	**Imputed cases**
	**(*N =* 1,965)**	**(*N =* 40 × 1,965)**
	**%**	**%**
**Gender**		
Boys	48.4	48.4
Girls	51.6	51.6
*Missing (n)*	–	–
**Current smoking at baseline**		
Yes	1.9	2.0
No	98.1	98.0
*Missing (n)*	(26)	–
**Current smoking at second follow-up**		
Yes	8.4	23.8
No	91.6	76.2
*Missing (n)*	(1,140)	–
**Ever use of ATPs at baseline**		
Yes	11.3	12.1
No	88.7	87.9
*Missing (n)*	(50)	–
**Ever use of ATPs at second follow-up**		
Yes	25.4	49.7
No	74.6	50.3
*Missing (n)*	(1,141)	–
**OSC**		
High	45.4	42.6
Medium	42.2	44.7
*Low*	12.4	12.6
*Missing (n)*	(443)	–

Table 3 shows the results of the difference-in-differences analyses of current smoking between socioeconomic groups. Adjusted for gender, the analyses of available cases showed that 0.3% of students from a high OSC currently smoked at baseline, while this was the case for 3.0% of students from a low OSC. At second follow-up, 8.3% of students from a high OSC and 10.9% of students from a low OSC currently smoked cigarettes. The adjusted difference-in-differences analysis of available cases showed an estimate close to zero, and the interaction term between OSC and time was insignificant. This indicates that there were no socioeconomic differences in smoking trajectories between OSC groups. However, the unadjusted and adjusted difference-in-differences analysis of imputed cases showed a somewhat different pattern. Adjusted for gender, 1% of the high OSC students were currently smoking at baseline, while this applied to 4.8% among students from a low OSC. At follow-up, 24.5% of the students from a high OSC and 25.6% from a low OSC were currently smoking.

**Table 3:** Difference-in-differences analyses of current smoking by occupational social class (OSC): analyses of available cases and imputed cases, unadjusted and adjusted for gender.

	**Current smoking at baseline**	**Current smoking at second follow-up**	***p*-value^**a**^**	**(C_**after**_-C_**before**_) –(E_**after**_-E_**before**_)^**b**^**
**Baseline cases**				
Unadjusted analysis (*n =* 690)	%	%		
High OSC	0.3	8.3	0.682	0.9
Medium OSC	1.3	7.4		
Low OSC	3.0	10.4		
Adjusted analysis (*n =* 642)	%	%		
High OSC	0.3	8.7	0.605	0.5
Medium OSC	1.4	7.4		
Low OSC	3.1	10.9		
**Imputed cases**				
Unadjusted analysis (*n =* 78,600)	%	%		
High OSC	1.0	24.4	<0.001	2.5
Medium OSC	2.1	22.6		
Low OSC	4.8	25.7		
Adjusted analysis (*n =* 78,600)	%	%		
High OSC	1.0	24.5		
Medium OSC	2.1	22.6	<0.001	2.7
Low OSC	4.8	25.6		

a *p-value of time x OSC interaction*.

b *difference-in-differences estimate (high vs. low OSC)*.

**Figure 2:**
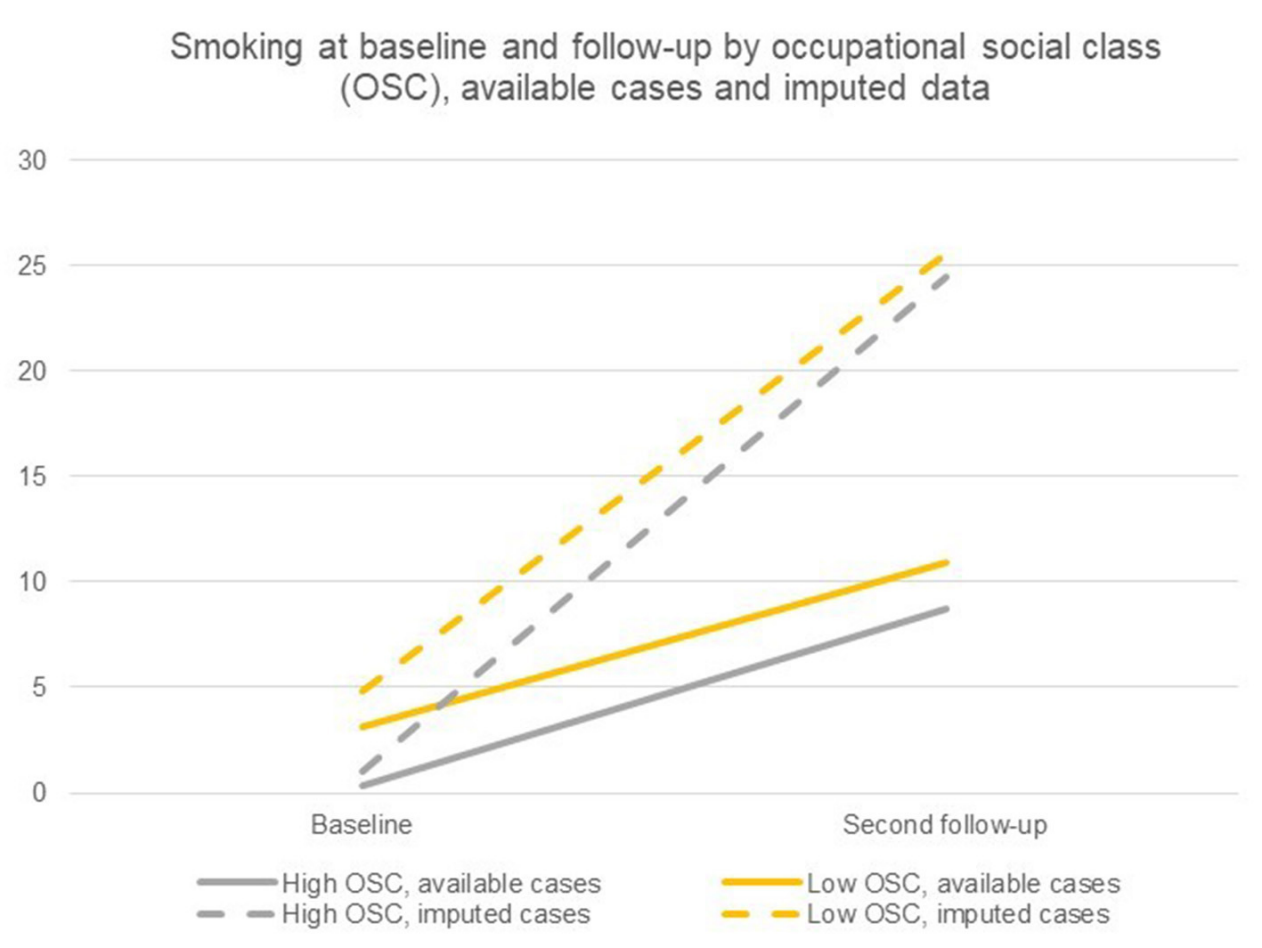
Current smoking at baseline and second follow-up by occupational social class (OSC), available cases and imputed cases.

Results showed that relative more students from a high OSC were currently smoking at second follow up relative to baseline compared with students from a low OSC (see also Figure 2); hence, indicating differential trajectories from baseline to second follow-up in current smoking among students from low and high OSC (*p* < 0.001).

Table 4 displays the difference-in-differences analyses of ever use of ATPs stratified by OSC of students. In adjusted analyses for gender of available cases, results showed that 7.6% of students from a high OSC and 11.6% from a low OSC had ever used ATPs at baseline. At second follow-up, 8.3% of students from a high OSC and 10.9% from a low OSC had ever used ATPs. The adjusted difference-in-differences analysis of imputed cases showed a similar pattern, although differences between OSC groups were more pronounced; adjusted for gender, 10.0% of students from a high OSC and 13.9% of students from a low OSC had ever used ATPs at baseline. At second follow-up, 46.8% of students from a high OSC and 60.8% of students from a low OSC had ever used ATPs.

**Table 4:** Difference-in-differences analyses of ever use of alternative tobacco products (ATPs) by occupational social class (OSC): analyses of available cases and imputed cases, unadjusted and adjusted for gender.

	**Ever use of ATPs at baseline**	**Ever use of ATPs at second follow-up**	***p*-value^**a**^**	**(C_**after**_-C_**before**_) –(E_**after**_-E_**before**_)^**b**^**
**Baseline cases**				
Unadjusted analysis (*n =* 690)	%	%		
High OSC	7.4	21.9	0.065	10.9
Medium OSC	11.5	24.7		
Low OSC	11.9	37.3		
Adjusted analysis (*n =* 642)	%	%		
High OSC	7.6	22.2	0.031	12.4
Medium OSC	12.3	25.1		
Low OSC	11.6	38.6		
**Imputed cases**				
Unadjusted analysis (*n =* 78,600)	%	%		
High OSC	10.0	46.8	<0.001	9.9
Medium OSC	13.8	49.5		
Low OSC	13.2	59.9		
Adjusted analysis (*n =* 78,600)	%	%		
High OSC	10.0	46.8	<0.001	10.1
Medium OSC	13.9	49.6		
Low OSC	13.9	60.8		

a *p-value of time x OSC interaction*.

b *difference-in-differences estimate (high vs. low OSC)*.

**Figure 3:**
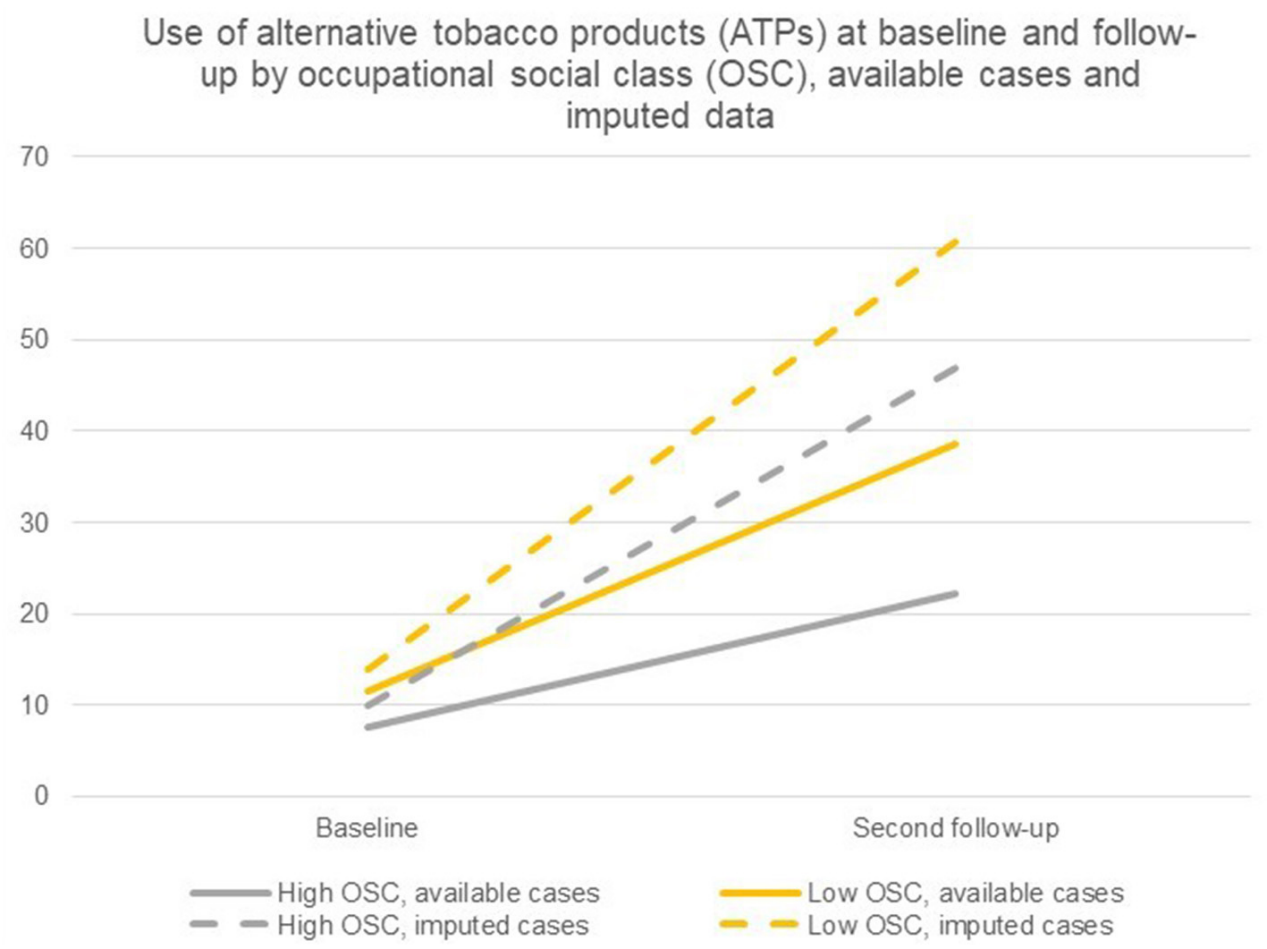
Ever use of ATPs at baseline and second follow-up by occupational social class (OSC), available cases and imputed cases.

The difference-in-differences estimates for the adjusted difference-in-differences analysis of available cases and imputed data indicated substantial differences in trajectories of ever use of ATPs between OSC groups (*p* < 0.001). Hence, relative more students from a low OSC had ever used ATPs at second follow up relative to baseline compared with students from a high OSC (see also Figure 3).

## Discussion

This study is among the first to examine differential trajectories of smoking and ATP use between socioeconomic groups before and after implementation of a smoking preventive intervention. Findings provide crucial knowledge for current and future actions against youth tobacco use, as initiatives should focus on benefiting all groups equally—or benefit the groups most in need of prevention efforts.

First, in line with most research (11), this study found that more students from low OSC were currently smoking cigarettes compared with adolescents from high OSC at baseline. However, from baseline to second follow-up, smoking developed somewhat differently among students from diverse socioeconomic backgrounds. Importantly, the gap in current smoking between students from low and high OSC narrowed over time from a difference of almost four percentage points at baseline to about one percentage point at second follow-up. In a recent study, we investigated trajectories of current smoking between OSC groups from baseline to first follow-up, i.e., at the end of grade 7 (52). The study indicated that X:IT II did not create differential trajectories in current smoking among adolescents from diverging socioeconomic backgrounds over the course of one school year. In the present study, students are followed until the end of grade 8 and thus, findings from the current study indicate that tobacco patterns have developed differently at this point in the intervention compared with the first follow-up measurement. Nevertheless, these findings should be viewed positively in the sense that the X:IT II intervention seems to be more beneficial for some groups of adolescents at higher risk of smoking cigarettes, i.e., from a low OSC. Thus, the intervention did not widen the social inequalities in smoking. Considering these findings, the X:IT II seems to be successful in targeting students from lower socioeconomic backgrounds. Similarly, a review by Tinner et al. (40) suggested that adolescents with lower socioeconomic positions benefited most from smoking preventive initiatives. However, there is generally a dearth of literature examining socioeconomic differences in the effect of smoking preventive interventions. The X:IT II intervention builds on a multilevel approach in which several components on individual and environmental levels are initiated to reduce adolescent smoking. This approach is considered ideal for limiting the social inequalities in intervention effects, while interventions solely aiming at the individual level may increase social inequalities (53).

From this study, we do not have knowledge about the development in smoking among adolescents unexposed to the intervention, although knowledge builds on previous findings from the first X:IT intervention with both an exposed and unexposed group. Some of the socioeconomic effects in smoking may have been impacted by an overall societal focus on decreasing smoking among youth, with increasing political actions against smoking (45). In this connection, one Danish study showed that the absolute socioeconomic differences in smoking decreased during the past decade; however, the relative socioeconomic differences in smoking increased during the same period, and smoking remained highest among students in the lowest socioeconomic group during the entire study period from 1991 to 2014 (11). Findings from other westernized countries also suggest rather stable or increasing socioeconomic differences in smoking over time (15, 54). As most political actions were in effect after implementation of this study (45), these initiatives were not expected to impact the study results. Future research may further investigate whether socioeconomic differences in smoking are influenced by the recent increased political and societal attention to smoking–these studies would also be fruitful for investigating whether trajectories in smoking varies over time for adolescents unexposed to intervention components.

Secondly, this study examined trajectories in ever use of ATPs between OSC groups. At baseline, more students from low OSC compared to high OSC had ever used ATPs. However, conversely to the development in current smoking during the study period, the socioeconomic differences in ever use of ATPs widened over time. There may be several mechanisms to explain these findings. First and foremost, the intervention was designed to prevent cigarette smoking uptake among students (44) and, thus, it was not a primary aim of the intervention to prevent the uptake of other tobacco products. Nonetheless, it has previously been discussed whether smoking preventive interventions may be effective in also reducing the use of ATPs as several characteristics associated with cigarette smoking are associated with ATP use (55). Consequently, many of the same mechanisms or pathways to cigarette smoking may apply to ATP use. However, a main focus on reducing cigarette smoking among adolescents may have some unfortunate side effects, e.g., pushing some groups of youths toward using other substances which may not be seen as just as harmful or damaging to health compared with conventional cigarettes. Our study may reflect this tendency. This may be due to the X:IT II intervention itself, which focused on cigarette smoking, combined with the current societal tendencies where laws on conventional cigarettes have been tightened substantially in recent years (45). The most recent adopted Danish law comprises several initiatives to prevent especially youth smoking and use of other tobacco and nicotine products, e.g., e-cigarettes, although these were not initiated at the time of the study. Moreover, in a recent study among students enrolled in the X:IT II intervention, students reported that knowing smoking is dangerous and not wanting to be addicted to smoking were the top reasons for not smoking cigarettes. These findings indicate a high awareness of the health-damaging effects of cigarette smoking (56). However, we do not have knowledge about students' reasoning toward using or not using ATPs.

The few studies that have evaluated the influence of tobacco prevention interventions on students' use of other tobacco products than conventional cigarettes have shown mixed results (57, 58). For example, an evaluation of an outdoor smoking ban found no impact on students' use of e-cigarettes and waterpipe at follow-up (57). In contrast Hedman et al. (58) found a lower prevalence of snus use among students in the intervention group compared with the control group. Here, the intervention comprised tobacco-free contracts and education in tobacco-related health issues. These findings indicate that interventions with multiple initiatives aiming at ATPs may potentially impact students' decisions about ATP use. The intervention components of X:IT II comprise parental involvement, including smoke-free agreements, as well as smoke-free school time, and smoke-free educational material. Thus, extending current intervention components to increase awareness and knowledge about ATPs may produce positive outcomes. However, none of the identified studies evaluated subgroup effects of the interventions in terms of students' socioeconomic backgrounds. As the use of ATPs is increasingly prevalent among adolescents, there is an urgent need for future research to examine the effect of prevention efforts on ATPs and whether the effect differs between socioeconomic groups.

### Methodological Considerations

The X:IT II intervention was based on a previously shown effective intervention in reducing smoking uptake evaluated in a large cluster-randomized controlled trial (36, 37). In this study, all schools were included as intervention schools, and the differential effects of the intervention between socioeconomic groups were evaluated utilizing a difference-in-differences design (44). Power calculations estimated a need for 48 schools which was fulfilled in the recruitment process where 57 schools agreed to participate. However, 11 schools withdrew from the study, leaving 46 schools enrolled in the baseline data collection. We were, nonetheless, very close to the calculated number of schools required for evaluating the X:IT II intervention. Further, schools enrolled in the X:IT II study are representative to Danish schools of schools nationwide in regard to organizational resources, enrolled number of students at schools, average grades, and students with foreign origin, although more public schools participated in the X:IT II study (59). To account for possible bias due to drop-out over time, we applied multiple imputation of data at second follow-up. In the imputation phase, 40 datasets were created with over- and underestimation of the school and class effects to account for intraclass correlations between school and classes. The social environment across schools as well as the intensity of the intervention may also vary between schools. However, as the aim of this study was to examine differential effects between students from high vs. low OSC, the differences between schools may not be that important in this specific study. We know that implementation most likely varies across schools—as was the case in the evaluation of the first X:IT intervention (60)—however, we do not expect implementation at the school level to be influenced by the individual level of OSC.

The two key assumptions behind the difference-in-differences approach are 1) parallel trends and 2) common shocks. It is thus assumed that parallel trends will occur between the exposed group and the comparison group before intervention and after the implementation of the intervention. As smoking is more common among students from low OSC compared with high OSC, this assumption was not fulfilled. Nonetheless, very few students smoke at the beginning of grade 7, and therefore, estimates for calculation of trends will be low and—at least to some extent—unreliable. The assumption of common shocks is that any event during or after the intervention will equally affect the exposure and comparison groups. This assumption is expected to be fulfilled as there is no reason to assume differences between the groups.

We used students' self-reporting of OSC which is a commonly used measure of students' socioeconomic backgrounds, i.e., it has been used in the Danish contribution to the WHO collaborative study “Health Behavior in School-aged Children study” for more than 20 years (11). The coding of OSC in Danish studies is adapted to the Danish labor market and is comparable to other measures utilized, e.g., the British Registrar General's social classification and the European socioeconomic classification (46, 61). The measure of OSC used in this study assesses both occupational skills and competencies necessary for the job as well as the power and control associated with the job position. Previous research has found that self-reports of parental occupation is a more valid measure of socioeconomic position than parental education (44). The measures used in this study to assess tobacco use among adolescents, i.e., current cigarette smoking and ever use of ATPs, respectively, were not directly comparable. Unfortunately, we did not have information about current use of ATPs. It could be that the intervention components were better suited for preventing current use more so than ever use, as the main outcome for the evaluation was current smoking. Thus, future research may further examine the impact of interventions on adolescents' current use of ATPs.

### Implications

Findings from the current study have several important implications. Specifically, findings suggest that the X:IT II intervention decreased socioeconomic inequalities in smoking over time. This is an important finding as adolescents from lower socioeconomic backgrounds are more at risk of using tobacco products (11). X:IT II was implemented in Danish lower secondary schools. The embeddedness in the school arena provides an ideal setting for reaching all children and adolescents regardless of their individual backgrounds. Therefore, the X:IT II intervention may be considered ideal for preventing smoking uptake among schoolchildren and adolescents. Nonetheless, this study also indicated that social inequalities in ever use of ATPs widened over time, with students from lower socioeconomic backgrounds being substantially more prone to have ever used these products. This may call for special attention in the design of preventive initiatives to account for the increasing use of and interest in ATPs among youth. As the current research on differential socioeconomic effects in tobacco preventive interventions is sparse, future research should be designed to address this lacking knowledge in subgroup effects of interventions. Moreover, a future area of research could be to examine the mechanisms in which socioeconomic differences in tobacco product use occur after the implementation of a tobacco preventive intervention.

## Conclusions

This study found that socioeconomic disparities in cigarette smoking narrowed over a 2 year-period in which a smoking preventive intervention (X:IT II) was implemented. X:IT II was designed to appeal equally to students from lower and higher socioeconomic backgrounds. Hence, this study indicates promising findings in impacting decisions about smoking among students from lower socioeconomic backgrounds. However, the current study found that social disparities in ever use of ATPs widened over the study period. As this study is among the first to examine differential trajectories of smoking and ATP use between socioeconomic groups before and after the implementation of a smoking preventive intervention, more research in this area is needed.

## Data Availability Statement

Data that support the findings of this available from the University of Southern Denmark (SDU). Restrictions to the availability of data that were used under license and are therefore publicly available. However, data are available from the corresponding upon reasonable request and with permission of SDU. Requests to access datasets should be directed to Simone Gad Kjeld, simk@sdu.dk.

## Ethics Statement

Ethical review and approval was not required for the study on human participants in accordance with the local legislation and institutional requirements. Written informed consent from the participants' legal guardian/next of kin was not required to participate in this study in accordance with the national legislation and the institutional requirements. However, parents of students received written information sent out by the schools and were verbally informed at a parents-teacher meeting about the purposes of this study and that participation of their child was voluntary. Further, students were verbally informed by the teachers before answering the questionnaires, and written information about the study was provided in the beginning of the questionnaire, including that students' participation was voluntary, and that their answers would be treated with confidence.

## Author Contributions

SK, LL, SA, and LB conceptualized and designed this study. LL carried out the main analyses. SK drafted the initial manuscript. LL, SA, and LB critically reviewed and revised the manuscript. All authors read and approved the final manuscript.

## Funding

This study was funded by the Danish Cancer Society. The Danish Cancer Society developed the intervention. The funding body was not involved in the design of the study, collection, analyses, interpretation of data, nor in the writing of the manuscript, and in the decision to submit the manuscript for publication.

## Conflict of Interest

The authors declare that the research was conducted in the absence of any commercial or financial relationships that could be construed as a potential conflict of interest.

## Publisher's Note

All claims expressed in this article are solely those of the authors and do not necessarily represent those of their affiliated organizations, or those of the publisher, the editors and the reviewers. Any product that may be evaluated in this article, or claim that may be made by its manufacturer, is not guaranteed or endorsed by the publisher.

## Abbreviations

OSC: Family occupational social class
ATPs: Alternative tobacco products.
